# Asynchrony adaptation reveals neural population code for audio-visual timing

**DOI:** 10.1098/rspb.2010.1737

**Published:** 2010-10-20

**Authors:** Neil W. Roach, James Heron, David Whitaker, Paul V. McGraw

**Affiliations:** 1 Visual Neuroscience Group, School of Psychology, The University of Nottingham, Nottingham, UK; 2 Bradford School of Optometry and Vision Science, University of Bradford, Bradford, UK

**Keywords:** auditory-visual timing, multisensory, population coding

## Abstract

The relative timing of auditory and visual stimuli is a critical cue for determining whether sensory signals relate to a common source and for making inferences about causality. However, the way in which the brain represents temporal relationships remains poorly understood. Recent studies indicate that our perception of multisensory timing is flexible—adaptation to a regular inter-modal delay alters the point at which subsequent stimuli are judged to be simultaneous. Here, we measure the effect of audio-visual asynchrony adaptation on the perception of a wide range of sub-second temporal relationships. We find distinctive patterns of induced biases that are inconsistent with the previous explanations based on changes in perceptual latency. Instead, our results can be well accounted for by a neural population coding model in which: (i) relative audio-visual timing is represented by the distributed activity across a relatively small number of neurons tuned to different delays; (ii) the algorithm for reading out this population code is efficient, but subject to biases owing to under-sampling; and (iii) the effect of adaptation is to modify neuronal response gain. These results suggest that multisensory timing information is represented by a dedicated population code and that shifts in perceived simultaneity following asynchrony adaptation arise from analogous neural processes to well-known perceptual after-effects.

## 1. Introduction

We typically perceive external events as coherent multi-sensory entities. When a balloon pops in front of us, for example, we *see* and *hear* it happen simultaneously. This is not trivial, given the considerable differences between the speed that light and sound travel through air, and the rate at which each is transduced into neural signals by our senses (see [1,2]). A flexible strategy the brain might employ to support accurate perception of timing is to monitor the temporal correspondence (e.g. cross-correlation) of sensory inputs and correct for pervasive delays between modalities. Studies demonstrating that our perception of simultaneity can be altered by recent experience are consistent with this active recalibration hypothesis. Short periods of adaptation to a consistent inter-modal asynchrony have been shown to shift an observer's point of subjective simultaneity (PSS) in the direction of the adapted asynchrony [3–12]. For instance, after exposure to sequences of auditory-visual stimuli in which the sound is consistently delayed, an auditory lag is typically required for subsequent stimuli to be perceived as simultaneous.

Traditional psychological models assume that perceived stimulus timing reflects the relative arrival time of sensory signals at some central brain site (e.g. [13–15]). Within this framework, situations in which synchronous sensory inputs give rise to asynchronous perception are most naturally interpreted as a consequence of disparate neural processing latencies. Indeed, findings that PSS estimates systematically deviate from zero have been taken as evidence for changes in processing latency as a function of visual field location [16], luminance [17], attentional state [18], stimulus feature (e.g. [19]) and sensory modality (e.g. [20]). In keeping with this approach, it has recently been proposed that changes in the PSS induced by auditory-visual asynchrony adaptation can be accounted for by an experience-dependent modulation of processing speed [21,22]. According to this hypothesis, processing of stimuli in one modality is accelerated or retarded during the course of adaptation to bring signals into temporal alignment with one another. Because such latency changes are inherently unisensory, a key prediction of this account is that adaptation to auditory-visual asynchrony should produce a uniform recalibration in which the perception of all audio-visual temporal relationships is altered equally.

**Figure 1.**
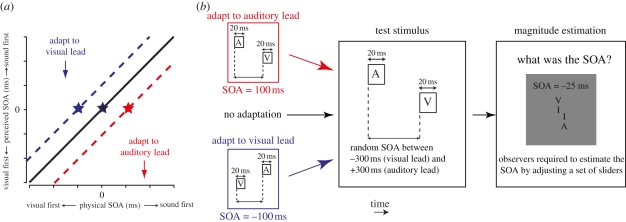
(*a*) Prolonged exposure to asynchronous auditory-visual stimuli alters the point of subjective simultaneity (PSS)—the physical stimulus-onset asynchrony required for stimuli to be perceived as simultaneous. Relative to baseline conditions with no adaptation (black star), the PSS shifts in the direction of the adapting asynchrony (red and blue stars). If this effect is representative of a uniform recalibration of perceived timing, the perception of all temporal relationships ought to be equally affected (diagonal lines). (*b*) Schematic of the experimental sequence, designed to measure adaptation-induced changes in perceived timing over a range of stimulus-onset asynchronies (SOAs). See main text for details.

Consider the recalibration process commonly performed when using a kitchen scale. Prior to measuring a quantity of flour, we first place an empty bowl on the scale and adjust the display to zero. This ‘zeroing’ of the kitchen scale is roughly analogous to the shifts in the PSS produced by asynchrony adaptation. In each case, we compensate for a potential source of error (the mass of the bowl or pervasive time delays) by adjusting the physical input (mass or asynchrony) required to produce null output (a reading of zero mass or the perception of simultaneity). The utility of kitchen scale calibration rests upon the fact that it applies a uniform, stimulus-independent correction for all subsequent measurements—regardless of how much flour we now add, we can be confident that the tare mass of the bowl will always be subtracted. A change in the processing speed in a given modality should operate in a functionally similar manner, exerting an effect that is independent of the temporal relationship between multimodal inputs. For example, if adapting to an auditory temporal lead over vision retards auditory latency by 30 ms, a 30 ms auditory lead will be perceived as close to synchronous, but a 100 ms auditory lead will also be perceived as closer to 70 ms. Thus, if mechanisms underlying asynchrony adaptation effects genuinely compensate for pervasive delays by adjusting processing latencies, one might expect resulting changes in perceived multisensory timing to be uniform in nature (figure 1*a*).

In this study, we measure the effects of adaptation to a fixed audio-visual asynchrony on the perception of a wide range of sub-second temporal relationships. In contrast to the uniform recalibration predicted by changes in sensory processing latency, we find that the magnitude of induced biases varies systematically as a function of the difference in stimulus-onset asynchrony (SOA) between adapting and test stimuli. To explain these findings, we consider an alternative working model of how the brain codes the relative timing of different sensory signals. The dominant coding strategy employed by the brain is to represent sensory information in the responses of specialized populations of neurons characterized by different tuning properties. Dedicated neural population codes have been characterized for numerous visual and auditory stimulus features, and significant progress has been made towards understanding the strategies employed by human observers when decoding this information to form perceptual decisions and plan actions (for recent reviews see [23–25]). Here, we develop a simple population-coding model of audio-visual timing that provides an excellent approximation of the varying effects of asynchrony adaptation. Our findings suggest that multisensory timing information may be represented in a fundamentally similar way to other sensory properties, and that shifts in the PSS following asynchrony adaptation arise from analogous neural processes to classic perceptual after-effects. Uniquely, however, the population code for multisensory timing may be characterized by intrinsic biases that arise as a consequence of the brain restricting neural representation to a finite range of audio-visual asynchronies.

## 2. Material and methods

### (a) Participants

Three of the authors served as participants, along with two adults who had experience of performing psychophysical tasks, but were naive to the specific purposes of experiment. Each had normal visual acuity and no history of hearing loss.

### (b) Stimuli

Visual stimuli were isotropic Gaussian blobs (*σ* = 2°) generated in Matlab and displayed via a Cambridge Research Systems ViSaGe on either a gamma-corrected Mitsubishi Diamond Pro 2045U or Sony Trinitron GDM-FW900 CRT monitor (mean luminance 47 cd m^−2^) at fixation for two video frames at 100 Hz. Auditory stimuli were 20 ms bursts of white noise (200 Hz—12 kHz passband, 5 ms cosine ramp at onset/offset), presented binaurally via Sennheiser HD-265 headphones. Auditory stimuli were convolved with a generic pair of head-related impulse response functions corresponding to a spatial position immediately in front of the observer (0° azimuth, 0° elevation; see [26] for measurement details).

### (c) Procedure

Although established methods exist for measuring shifts in the PSS following asynchrony adaptation, quantification of adaptation-related changes in perception across a range of SOAs poses more of a methodological challenge. Participants typically have a robust concept of what is meant by ‘simultaneous’, providing an internal standard against which stimuli can be judged (e.g. as in synchronous/asynchronous or temporal order judgements). However, because strong internal standards are not available for different temporal relationships (try to imagine a visual stimulus leading an auditory one by 170 ms, for example), such single-interval binary judgements are ill-suited to the measurement of perceived temporal relationships across a broad range of asynchronies. The obvious alternative is to pair test stimuli with an explicit standard stimulus with a fixed SOA (i.e. a two-alternative forced choice procedure). However, this approach is also problematic in this instance, because the perceived timing of the standard stimulus will also be affected by adaptation. To circumvent these problems, we opted to use magnitude estimation, a classical psychophysical procedure most often associated with Stevens's pioneering work on brightness and loudness perception (e.g. [27]). As depicted in figure 1*b*, participants were required to estimate the SOA between pairs of brief auditory and visual stimuli with and without prior adaptation to a fixed asynchrony (100 ms visual-lead or 100 ms auditory-lead). Adaptation consisted of 120 initial presentations of the asynchronous audio-visual pair, plus four additional top-up presentations prior to each test stimulus. To obviate exposure to a consistent unimodal timing pattern during adaptation, the interstimulus interval between successive audio-visual pairs was randomly jittered in the range 400–600 ms. The SOA of each test stimulus was sampled (with replacement) from a uniform distribution spanning −300 ms (visual lead) to +300 ms (auditory lead). Participants were required to indicate the perceived SOA of the test pair via a graphical user interface comprising a scaled schematic of the time-course and numerical SOA value that could be adjusted to 5 ms precision. The initial SOA indicated by the graphical-user interface was randomized to disperse the effect of any potential biases related to the starting position of the adjustment process. Practice was provided at the beginning of each experimental session to familiarize participants with the extremes of the SOA range (±300 ms). Participants were informed that the SOA of all stimuli was restricted to this range. All participants first completed a number of experimental sessions for the baseline condition with no adaptation, before completing sessions with adaptation to an auditory visual lead in a randomized order.

### (d) Modelling

We began by assuming that the temporal relationship between auditory and visual signals is represented across a population of *N* neurons tuned to different SOAs. The tuning function *f* of each neuron was described by a Gaussian function of the form
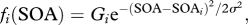
where *G*_*i*_ and SOA_*i*_ are the response gain and preferred SOA of the *i*th neuron, respectively, and *σ* sets the width of the tuning function (common to all neurons). Tuning functions were distributed uniformly around physical synchrony with a fixed 50 ms separation. Adaptation was modelled as a reduction in response gain, the magnitude of which falls off as a Gaussian function of the difference between adapted (SOA_*a*_) and preferred (SOA_*i*_) asynchronies
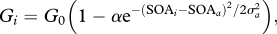
where the unadapted response gain *G*_0_, maximal proportional gain reduction *α* and breadth of the gain field (*σ*_*a*_) were common to all neurons.

The response *R*_*i*_ of each neuron to a test stimulus on any given trial was determined from its tuning curve and corrupted by independent Poisson noise such that
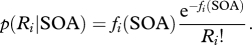


To decode the noisy population response, the log likelihood of each potential SOA was calculated (see [28]) as
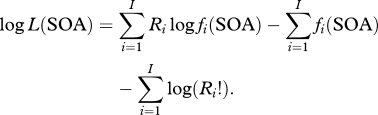


The SOA with the maximum log-likelihood was then taken as the model's estimate on that trial. In order to produce biases in perceived timing, it was assumed that the maximum-likelihood decoder was ‘unaware’ of the effects of adaptation (i.e. the log-likelihood calculation used the unadapted tuning function *f*_*i*_(SOA); see [29]).

## 3. Results

**Figure 2.**
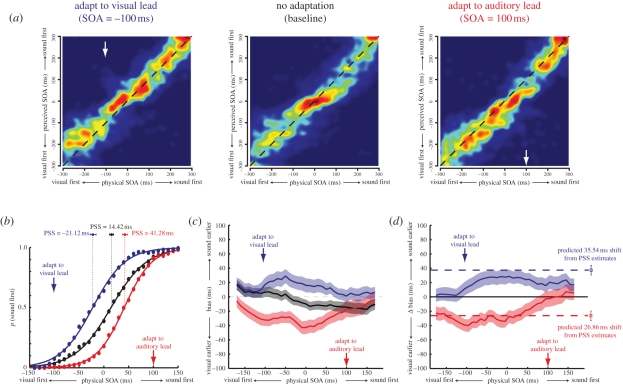
(*a*) Image maps representing the distribution of estimates at each physical stimulus-onset asynchrony (SOA). ‘Warmer’ colours indicate higher probabilities. To aid clarity, the observed values have been convolved with an isotropic Gaussian smoothing filter (*σ* = 20 ms). The dashed black lines indicate veridical estimation. (*b*) Reconstructed psychometric functions for temporal order discrimination. The probability of perceiving the auditory stimulus as leading the visual stimulus is plotted as a function of SOA. Solid lines show the best-fitting logistic functions for each condition (colour coding as shown in figure 1). Clear evidence can be seen for a shift in the point of subjective simultaneity (PSS) towards the adapted asynchrony. (*c*) Biases of perceived timing induced by asynchrony adaptation. The mean bias (difference between physical and estimated SOA) is plotted as a function of SOA for each condition. (*d*) Shifts in mean bias from baseline are shown across the range of sampled SOAs. For comparison, the dashed horizontal lines indicate the pattern of results that would be expected if PSS shifts were representative of a uniform recalibration of perceived timing. In this and subsequent figures, shaded regions indicate the 95% confidence intervals.

Estimates were collated across observers (approx. 7000 total trials). Image maps representing the distribution of perceived SOA estimates as a function of physical SOA are shown in figure 2*a*. In this and subsequent figures, negative and positive SOA values indicate visual and auditory leads, respectively. In each condition, physical and perceived SOAs were highly correlated (*r*_visual-lead_ = 0.92, *r*_no-adaptation_ = 0.88, *r*_auditory-lead_ = 0.91), indicating that observers were able to form estimates with a reasonable degree of precision. To avoid potential problems encountered at the extremes of the sampled SOA interval (e.g. ‘clipping’ of estimates that would have fallen outside the range), subsequent analysis was restricted to the range between −200 ms and +200 ms. Trials were binned according to the sampled physical SOA (60 ms bin width, 10 ms centre-to-centre bin separation). Instances in which estimation error (SOA_perceived_ − SOA_physical_) was more than 3 s.d. away from the bin mean were removed, accounting for less than 3 per cent of all estimates.

### (a) Shifts in the point of subjective simultaneity

In order to facilitate comparison with the pre-existing accounts of asynchrony adaptation, we first used the polarity of non-zero-perceived SOA estimates (i.e. whether it was auditory-first or visual-first) to reconstruct psychometric functions for temporal order discrimination. Figure 2*b* shows the probability of perceiving the auditory stimulus to lead the visual stimulus, plotted as a function of the physical SOA. Solid lines show the best-fitting logistic function
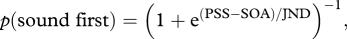
where PSS is defined as the physical SOA at which participants are equally likely to judge the auditory stimulus as leading or lagging and JND is an index of the discrimination threshold (the just-noticeable difference). Consistent with previous studies measuring explicit (i.e. binary) temporal order judgements (e.g. [4,9]), we found that audio-visual asynchrony adaptation systematically shifted the PSS towards the exposed asynchrony (PSS_visual-lead_ = −21.12 ms, PSS_no-adaptation_ = 14.42 ms, PSS_auditory-lead_ = 41.28 ms), and that discrimination thresholds were similar in the three conditions (JND_visual-lead_ = 32.56 ms, JND_no-adaptation_ = 35.93 ms, JND_auditory-lead_ = 27.76 ms).

### (b) Non-uniform changes in perceived auditory-visual timing

The advantage of our experimental approach is that it permits us to carry out a more detailed analysis of perceived timing. To quantify the biases in the perception of different temporal relationships, we next calculated the difference between physical and perceived SOA values on each individual trial. Figure 2*c* displays mean biases in each condition, plotted as a function of the test stimulus SOA. Shaded regions indicate the 95 per cent confidence intervals calculated using non-parametric bootstrapping [30]. One point of note is that rather than being horizontal, the bias function for the unadapted condition (shown in black) has a negative slope. This suggests a compressive bias—on average, asynchronous auditory-visual stimuli are judged to be slightly less asynchronous than they actually are. We will return to this point in a subsequent section.

If asynchrony adaptation acts to induce a uniform recalibration of the perceived audio-visual timing, bias profiles for each adaptation condition should resemble a vertical translation of that obtained in the no-adaptation condition. Contrary to this prediction, however, changes in bias are highly non-uniform across the sampled SOA interval. Whereas adaptation induced large, statistically significant shifts in bias for certain SOAs, others remained indistinguishable from baseline. This departure from uniformity can be clearly seen in figure 2*d*, which plots the difference in bias between the adapting and baseline conditions. Results are poorly approximated by the horizontal dashed lines, which designate the pattern of results that would be expected if measured shifts in the PSS were representative of a uniform recalibration of audio-visual timing perception. Rather, the magnitude of induced biases appears to increase as the SOA of the test stimulus is moved away from that of the adaptor. These findings are inconsistent with the operation of a mechanism that compensates for an adapted auditory-visual asynchrony by adjusting the speed of processing in one or both modalities.

### (c) Characterizing adaptation-induced biases with a population-coding model

**Figure 3.**
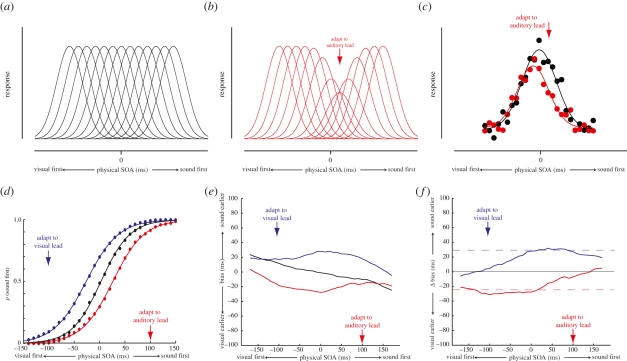
Modelling the effects of auditory-visual asynchrony adaptation. (*a*) Schematic of a population code comprising neurons tuned to different auditory-visual stimulus-onset asynchronies (SOAs). In the best-fitting model, there were a total (*n*) of 29 neurons and each had a Gaussian tuning profile with an s.d. (*σ*) of 220.60 ms. (*b*) The effects of adaptation were modelled as a selective reduction of response gain around the adapted SOA. Best-fitting parameter values were a maximum proportional gain reduction (*α*) of 0.41 and a gain field standard deviation (*σ*_*a*_) of 122.61 ms. See §2*d* for further details. (*c*) Examples of population responses to a physically synchronous auditory-visual stimulus (SOA = 0 ms). Data points represent individual noisy neuronal responses, plotted as a function of their preferred SOA. Asynchrony adaptation produces a repulsive shift of the population response profile away from the adapted SOA. (*d*) Psychometric functions for temporal order discrimination reconstructed from the simulated dataset, demonstrating the resulting shift in the point of subjective simultaneity. (*e*) Mean bias of SOA estimates in the simulated dataset as a function of SOA. (*f*) Shifts in mean bias from baseline for each of the adaptation conditions in the simulated dataset.

To explain the non-uniform effects of asynchrony adaptation, an understanding of how the brain encodes the relative timing of multisensory events is required. At present, however, the nature of the mechanisms involved is unclear. One potential solution might be to represent relative time via the pattern of activity across a population of neurons tuned to different inter-sensory delays (e.g. [3,31]). An appeal of this approach is that shifts in the PSS following asynchrony adaptation could be viewed as an analogue of well-documented visual after-effects in the orientation (e.g. [32]) and motion (e.g. [33]) domains. Extant models of these sensory after-effects posit that adaptation selectively reduces the gain of neurons tuned to the adapted stimulus, resulting in a repulsive shift of the population response to subsequent stimuli away from the adapted value (e.g. [34,35]). Figure 3*a*–*c* illustrates how a comparable population-coding model might explain changes in perceived simultaneity following adaptation to asynchronous auditory-visual stimuli.

To test this approach in a quantitative manner, we carried out a series of trial-by-trial simulations in which we repeated the asynchrony adaptation experiment while replacing the responses of the psychophysical observers with the output of the population-coding model (see §2*d* for details). Each simulation comprised 10 000 trials and the resulting dataset was analysed in an identical manner to the empirical study. Across successive simulations, four parameters were free to vary: the number of neurons in the population (*N*), the bandwidth of their tuning (*σ*), and the depth (*α*) and bandwidth (*σ*_*a*_) of the gain reduction induced by adaptation. These model parameters were optimized so as to minimize the squared residual error between the adaptation-related change in bias produced in the empirical (i.e. figure 2*d*) and simulated experiments. Results for the best-fitting model are shown in figure 3*f*, which successfully accounted for approximately 94 per cent of the variance in the original dataset. In addition, the model produced accurate approximations of the reconstructed psychometric functions for temporal order discrimination (figure 3*d*) and the bias profiles for each individual condition (figure 3*e*). In contrast to the uniform recalibration predicted by a change in sensory processing latency, this population-coding approach is clearly able to capture the non-uniform effects of asynchrony adaptation.

### (d) Compressive biases and neural population codes with a finite range

**Figure 4.**
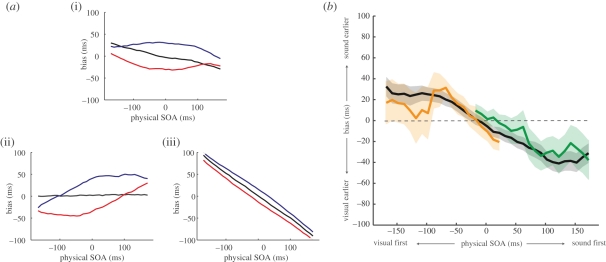
(*a*) Effect of changing the absolute number of neurons in the model population code. (i) Predicted patterns of bias of the best-fitting model, comprising 29 neurons with SOA preferences ranging from −700 ms to +700 ms. Evidence of a compressive bias can be seen in the unadapted baseline condition (black line). (ii) Increasing the range of preferred SOAs to ±2000 ms (81 neurons) while keeping all other factors constant removes the compressive bias from the baseline condition. (iii) Conversely, reducing the range of representation to ±500 ms (21 neurons) results in a magnification of the compressive bias. (*b*) Compression occurs around the point of simultaneity, rather than the centre of the response range. The black line shows mean biases in the unadapted condition for three observers, where the SOA of test stimuli was randomly drawn from a uniform distribution centred on physical synchrony (0 ms SOA). Comparable patterns of bias were obtained when the range of potential SOAs was offset by 150 ms (green line) or −150 ms (orange line), indicating that the locus of the compressive bias is an SOA near synchrony, not the centre of the response range. Shaded regions indicate the 95% confidence intervals.

In the model, we derived each SOA estimate from the neural population response using a maximum-likelihood decoder (see §2*d*). This read-out strategy is regularly employed in the literature because it is often ‘optimal’, providing estimates that are unbiased and with the lowest possible variance (e.g. [28,36,37]). With this in mind, an unexpected outcome of the simulations was that even in the absence of adaptation, estimates were systematically biased. As shown by the black line in figure 3*e*, the model underestimated the magnitude of asynchronous stimuli in the unadapted condition, reproducing the compressive bias previously noted in the baseline condition of the empirical experiment. The reason for this is that the optimality of the maximum-likelihood decoder can break down when there are relatively small numbers of neurons in the population code (see [36]). This effect is illustrated in figure 4*a*, which shows patterns of bias produced by variants of the population-coding model characterized by different ranges of tuning preferences. Figure 4*a*(i) shows results for the best-fitting model, which comprised 29 neurons with preferred SOAs ranging from −700 ms to +700 ms. Increasing the range of representation by adding additional neurons to the population abolishes the compressive bias in the baseline condition (figure 4*a*(ii); 81 neurons, preferred SOA range −2000 ms to +2000 ms), whereas reducing the range by removing neurons produces a pattern of results dominated by the compressive bias (figure 4*a*(iii); 21 neurons, preferred SOA range −500 ms to +500 ms). Importantly, a change in the number of neurons in either direction produces a pattern of results in baseline and adaptation conditions that is a poorer fit of the empirical dataset. Note that a comparable pattern of results can also be simulated by varying the spacing of preferred SOAs in a population with a fixed number of neurons, suggesting that the critical factor is the range of preferred SOAs, rather than the number of neurons *per se*.

An intriguing possibility raised by these results therefore is that the compressive bias displayed by participants might be indicative of the use of an inherently inaccurate population code. Before we can conclude this, however, it is necessary to consider an alternative explanation of this effect. In principle, measured biases could simply reflect an artefact of observers not using the full range of SOAs in the estimation task. Response range compressions have been documented to occur in some magnitude estimation paradigms, and are classically referred to as ‘regression effects’ (see [38,39]). A critical difference between these explanations is that whereas the small sample bias account predicts a compression of perceived SOA around a fixed point (the centre of the range of neuronal preferences in the population code), a regression effect should be linked to the particular response range in the estimation task. To dissociate between these different accounts, we had a subset of observers repeat the baseline (no adaptation) condition while offsetting the range of potential SOAs by ±150 ms (i.e. instead of −300 ms to +300 ms, the range was −150 ms to +450 ms, or −450 ms to +150 ms). To avoid any net adaptation to a particular temporal order in a run of trials, these two conditions were interleaved with one another—the test stimulus was randomly drawn from the range centred on +150 ms for odd trial numbers, and the range centred on −150 ms for even trial numbers. All other methods were identical to those described previously. Results are shown in figure 4*b*. If regression to the centre of the response range were occurring, we would expect bias profiles in these offset conditions to be shifted along the horizontal axis. Contrary to this prediction, however, patterns of bias were very similar to those found in the original measurements. Perceived SOAs are compressed around a consistent point somewhere near physical synchrony, rather than around the centre of the particular response range imposed on the observer. Accordingly, it appears unlikely that a simple response bias artefact of this kind can account for these biases.

### (e) Response gain and estimate variability

**Figure 5.**
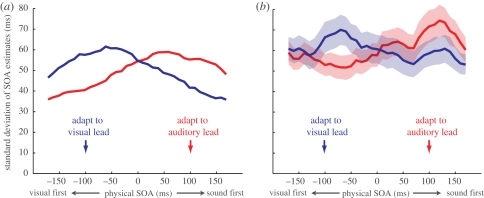
Patterns of estimate variability in the simulated and empirical datasets. The standard deviation of binned SOA estimates is shown following adaptation to a visual lead of 100 ms (blue lines) or an auditory lead of 100 ms (red lines). (*a*) In the model, selective reduction of neuronal response gain leads to more variable estimates of SOAs that are of the same polarity as the adaptor than those that are of opposite polarity. (*b*) This pattern is also evident in the empirical dataset.

In order to approximate the perceptual biases induced by exposure to asynchronous stimuli, we have modelled adaptation as a selective change in neuronal response gain. Rather than solely producing bias, population-coding theory dictates that these physiological changes ought to also affect the variability of estimates (e.g. see [29,40]). To investigate this possibility, we computed the standard deviation of estimates within each SOA bin in the simulated and empirical datasets. Figure 5*a*,*b* shows the results for each adaptation condition derived from the model and participant estimates, respectively. Although there is a difference in terms of absolute level of variability, both plots clearly show a similar pattern. Estimates of SOAs that are of the same polarity as the adapting stimulus are more variable than those of opposite polarity. It is important to note here that although the parameters of the model were optimized to fit the patterns of bias in the empirical dataset, the fitting procedure itself did not take into account estimate variability.

## 4. Discussion

After exposure to a consistent temporal delay between auditory and visual stimuli, perceived auditory-visual simultaneity is adjusted to compensate for the adapted lag. Recent proposals that this phenomenon arises as a consequence of changes in perceptual processing latency [21,22] predict a uniform recalibration, in which the perception of all auditory-visual temporal relationships is equally affected. Contrary to this prediction, however, in the present study we have shown that changes in perceived timing induced by asynchrony adaptation vary systematically as a function of the difference between adapted and tested SOAs. This finding is difficult to reconcile with any explanation based on sensory processing changes within either (or both) modalities. Instead, it suggests that asynchrony adaptation acts upon representations of the temporal relationship between auditory and visual inputs itself.

How does the brain represent the relative timing of different sensory inputs? Here we propose that, like many basic unisensory properties, multisensory timing is at some level represented by a dedicated population code comprising neurons tuned to different asynchronies. Multimodal neurons exhibiting broad selectivity for particular auditory-visual temporal relationships have previously been reported in subcortical and cortical areas (e.g. [41–44]). These neurons are typically viewed as the foundations of a temporal window of integration, within which sensory signals are likely to be bound together and perceived as arising from a common source. However, as our simulations demonstrate, the response profile of a population of such neurons is also an information-rich code capable of supporting the discrimination of a range of different auditory-visual temporal relationships. One issue for a dedicated population code of relative multisensory timing is setting the range of representation. For circular variables, such as the orientation or direction of motion of a visual stimulus, a population code can uniformly tile the space of all potential values. In contrast, representation of all potential temporal relationships between sensory inputs would require an infinite set of neural detectors. The system must therefore strike a balance between the range of representation of the population code and the allocation of neural resources. An interesting outcome of our modelling was that the optimization procedure converged upon a population of neurons with preferences spanning a finite range of SOAs (±700 ms). Such a range is likely to be sufficient to permit representation of most behaviourally relevant auditory-visual temporal relationships (i.e. those relating to common or causally linked events in the environment). However, the disadvantage of having a relatively small number of detectors is that the population code becomes inherently biased. Indeed, both the experimental and simulated datasets displayed evidence of a systematic compressive bias, in which SOA estimates were shifted towards synchrony. Therefore, it is possible that in representing the temporal relationship between auditory and visual stimuli the brain is forced to sacrifice perceptual accuracy to limit the overall metabolic demands. Although population-coding approaches have previously been employed in a variety of sensory neuroscience applications (for recent reviews see [25,40]), as far as we are aware this is the first time in which human performance has been successfully modelled using an intrinsically biased population code.

Although our experiments focused exclusively on auditory-visual timing, it is likely that our results have wider implications for the representation of temporal relationships in the brain. Shifts in perceived simultaneity have also been demonstrated following adaptation to asynchronous auditory-tactile [4] and visual-tactile [4,10,45] stimuli, suggesting that it is likely that common processing strategies operate across different sensory combinations. Moreover, it has recently been shown that transfer of adaptation effects between different bimodal pairings can occur under some circumstances [22], raising the possibility that some overlap might exist between representations of the temporal relationship between different sensory inputs. Interesting parallels also exist between our results and previous findings in studies of sensory-motor timing perception. In 2002, Haggard *et al.* [46] demonstrated that subjects consistently underestimate the temporal delay between a voluntary motor act (a key press) and a subsequent sensory event (an auditory tone). This systematic bias, which they termed ‘intentional binding’, is similar in nature to the compressive bias we report for auditory-visual stimuli. Coupled with the finding that perceived simultaneity can also be manipulated by exposure to a fixed delay between actions and sensory consequences [47–49], this result suggests that the combination of intrinsic and adaptation-induced biases reported in the present study are mirrored in the sensory-motor domain.

In our model, asynchrony adaptation was implemented by selectively reducing the response gain of audio-visual neurons. Selective response suppression is the most commonly reported physiological consequence of adaptation (see [50]), and has long been considered the primary contributor to repulsive perceptual after-effect phenomena (e.g. [51–53]). Using this approach, we demonstrate that it is possible to successfully capture the non-uniform pattern of biases in perceived auditory-visual timing following asynchrony adaptation. Moreover, the model also made predictions about the relative precision of timing estimates in adapted conditions that were borne out in the experimental dataset. It is important to note that this simultaneous characterization of bias and variability could not be achieved if the gain control mechanism was replaced with a different form of plausible adaptation effect, such as the modification of tuning width or a shift in the tuning preferences of the underlying neuronal population (see [29,40]). Although our data cannot rule out that such changes might contribute in some way, it does strongly suggest that response suppression is the primary mechanism driving asynchrony adaptation effects. As such, our results provide clear predictions for future physiological studies investigating the effects of asynchrony adaptation on the responses of multimodal neurons.

Application of a population-coding approach to multisensory timing provides a parsimonious explanation of the effects of asynchrony adaptation. Within this framework, changes in perceived simultaneity arise from computationally similar processes to classic sensory adaptation phenomena such as the tilt after-effect (e.g. [32]) and the direction after-effect (e.g. [33]). Given this similarity, it is interesting to consider how markedly different the interpretations of the broader functional significance of these effects are. Although the precise functional role of sensory adaptation remains a topic of active debate, it is generally agreed that its ultimate purpose is to improve the efficiency of neural coding, and that perceptual biases arise as a side-effect of the process. In contrast, the notion that asynchrony adaptation reflects a temporal recalibration mechanism supposes that induced biases in perceived timing are the primary functional outcome. While it is not inconceivable that computationally similar neural processes might support different functional outcomes, in the future it might pay to consider sensory and multisensory adaptation effects within a common theoretical framework.
